# Revisiting Hemicraniectomy: Late Decompressive Hemicraniectomy for Malignant Middle Cerebral Artery Stroke and the Role of Infarct Growth Rate

**DOI:** 10.1155/2017/2507834

**Published:** 2017-03-16

**Authors:** Saadat Kamran, Naveed Akhtar, Abdul Salam, Ayman Alboudi, Kainat Kamran, Arsalan Ahmed, Rabia A. Khan, Mohsin K. Mirza, Jihad Inshasi, Ashfaq Shuaib

**Affiliations:** ^1^Neuroscience Institute (Stroke Center of Excellence), Hamad Medical Corporation, Doha, Qatar; ^2^Weill Cornell School of Medicine, Doha, Qatar; ^3^Rashid Hospital, Dubai, UAE; ^4^College of Liberal Arts and Sciences, University of Illinois at Chicago, Chicago, IL, USA; ^5^Shifa International Hospital, Islamabad, Pakistan; ^6^Stroke Program, University of Alberta, Edmonton, AB, Canada

## Abstract

*Objective and Methods.* The outcome in late decompressive hemicraniectomy in malignant middle cerebral artery stroke and the optimal timings of surgery has not been addressed by the randomized trials and pooled analysis. Retrospective, multicenter, cross-sectional study to measure outcome following DHC under 48 or over 48 hours using the modified Rankin scale [mRS] and dichotomized as favorable ≤4 or unfavorable >4 at three months.* Results.* In total, 137 patients underwent DHC. Functional outcome analyzed as mRS 0–4 versus mRS 5-6 showed no difference in this split between early and late operated on patients [*P* = 0.140] and mortality [*P* = 0.975]. Multivariate analysis showed that age ≥ 55 years, MCA with additional infarction, septum pellucidum deviation ≥1 cm, and uncal herniation were independent predictors of poor functional outcome at three months. In the “best” multivariate model, second infarct growth rate [IGR2] >7.5 ml/hr, MCA with additional infarction, and patients with temporal lobe involvement were independently associated with surgery under 48 hours. Both first infarct growth rate [IGR1] and second infarct growth rate [IGR2] were nearly double [*P* < 0.001] in patients with early surgery [under 48 hours].* Conclusions.* The outcome and mortality in malignant middle cerebral artery stroke patients operated on over 48 hours of stroke onset were comparable to those of patients operated on less than 48 hours after stroke onset. Our data identifies IGR, temporal lobe involvement, and middle cerebral artery with additional infarct as independent predictors for early surgery.

## 1. Introduction

The issue of optimal timing of decompressive hemicraniectomy [DHC] in patients with malignant middle cerebral artery (MMCA) stroke has not been satisfactorily addressed by the randomized trials and pooled analysis [1–4]. In systematic reviews, the time to surgery had no significant relation to outcome [5, 6]. Moreover, the pooled analysis of European trials did not demonstrate any benefit of early DHC before 24 hours versus later surgery [3]. With the exception of systematic review [6] (HAMLET and HeADDFIRST [11 patients in HAMLET and 8 patients in HeADDFIRST]), patients included in the randomized trials and their pooled analyses were treated within 48 hours of symptom onset. A number of small prospective observational and retrospective studies have included patients operated on after 48 hours [6–16] but the number of patients is small to draw any definitive conclusion.

Randomized controlled trials in DHC are difficult to conduct due to ethical considerations of high mortality in control groups and slow patient recruitment. Pooled databases may be able to provide additional information regarding the aforementioned question. The purpose of this multi-institutional pooled data analysis from three countries was to determine the impact of DHC timing on the functional outcomes and identify prognostic factors in patients operated on beyond 48 hours in comparison with DHC under 48 hours. In addition, factors leading to early or late DHC were also identified. The rate of infarct growth varies from patient to patient and the clinical implication of the infarct growth rate [IGR] in deciding timing of DHC has received little attention in the literature. We hypothesized that the primary reason for early surgery may be the faster infarct growth that leads to symptom progression and hence earlier surgery.

## 2. Patients and Methods

A retrospective DHC database from three tertiary referral centers in three countries [Hamad General Hospital, Qatar; Rashid Hospital, Dubai, UAE; and Shifa International Hospital, Pakistan] collected over a period of eight years, between 2007 and 2014, was analyzed. The DHC database included all patients referred for surgery based on the following criteria: National Institutes of Health Stroke Scale [NIHSS] score ≥ 16 including a score of 1 for item 1a (decreased level of consciousness) from the beginning or progressive deterioration within 24–48 hours, brain computed tomography [CT] evidence of ischemia involving two-third middle cerebral artery [MCA] or 50% MCA with additional anterior cerebral artery [ACA] or posterior cerebral artery [PCA] infarction, and signs of local swelling [effacement of the sulci, compression of the lateral ventricle]. Patient's clinical records were reviewed for demographics, risk factors (hypertension, diabetes, hyperlipidemia, smoking, and coronary artery disease), time of stroke onset, time and type of imaging studies, medical treatment including intensive care stay, hyperosmolar therapy, signs of herniation, time to herniation, and time to surgery. The NIHSS and the Glasgow Coma Score (GCS) were used to assess the severity of the neurological deficit at the time of admission and deterioration. Radiological analysis included measurement of the infarct volume [IV] using *ABC*/2, where *A* is the largest cross-sectional diameter, *B* is the largest diameter 90° to *A* on the same slice, and *C* is the approximate number of CT slices on which the stroke is seen and divided by 2 to approximate the volume of an ellipsoid [17]. The method is simple and has been validated previously for ischemic stroke [17].

Maximum infarct volume [MIV] was calculated using last CT before DHC.

For the type of vessel occlusion, CT angiography (CTA), MR Angiography (MRA), or a conventional digital angiogram was utilized.

For measurement of septum pellucidum shift, a straight line was drawn in the expected location of the septum pellucidum from the most posterior aspects to the falx on axial images. Shift of the septum pellucidum from this midline was measured and compared to subsequent CT scans to determine any change.

The decision regarding the time of surgery was at the discretion of the treating physician/surgeon. Patients were generally taken for surgery if there was deterioration in the level of consciousness with clinical signs of herniation.

Patients were excluded if significant contralateral infarction or preexisting infarction was present on the initial CT, if only a single imaging study was performed or if imaging was uninterruptable, with parenchymal hematoma grade II [18], hemorrhage causing clinical deterioration, or hemorrhage with ventricular extension, missing surgical details, and if the 3-month follow-up data was not available.

Outcome was assessed with the modified Rankin score (mRS) dichotomized as favorable [mRS ≤ 4] and unfavorable [mRS > 4] at three months by patient examination in the outpatient clinics. We also looked at the outcome in subgroup stratified by time [<24, 24–<48, 48–72, and >72 hours] to DHC.

## 3. Infarct Growth Rate Calculation

For first infarct growth rate [IGR1] calculation, we assumed the stroke volume to be zero prior to stroke onset.

(1) $$\begin{array}{c} IGR1=\frac{\Delta \;\;volume\;\;\left(infarct\;\;volume\;\;CT1-0\right)}{\Delta \;\;time\;\;\left(time\;\;CT1-stroke\;\;onset\;\;time\right)}. \end{array}$$

Second infarct growth rate [IGR2] was measured on second CT [CT2] using the following formula:

(2) IGR2=Δ  volume  infarct  volume  CT2−infarct  volume  CT1Δ  time  time  CT2−time  CT1.

The hospitals included in the study are tertiary referral centers accredited by joint commission international. They are affiliated with medical schools and have residency-training programs. A well-established comprehensive stroke service including acute stroke diagnostic, vascular interventional services, stroke units, and rehabilitation services is in place. An acute stroke team provides a rapid assessment service 24 hours a day, seven days a week. Each hospital has a neurological surgery program, actively participating in vascular neurology service.

## 4. Data Analysis Plan

All statistical analyses were performed using Statistical Package for Social Sciences Version 22 (SPSS). Descriptive and inferential statistics were used to characterize the study sample and test hypotheses. Descriptive results (including graphical displays) for all quantitative variables (e.g., age) are presented as mean ± standard deviation (SD) (for normally distributed data) or median with interquartile range (for data not normally distributed). Numbers (percentage) were reported for all qualitative variables (e.g., gender). Bivariate analysis was performed using independent sample *t*-test or Mann–Whitney *U* test whenever appropriate to compare all quantitative variables (e.g., age) between those who underwent surgery within 48 hours and those whose surgery was done beyond 48 hours from the time of onset of symptoms. All of the qualitative variables (e.g., gender and HTN) between two groups (DHC ≤ 48 versus > 48) were compared using Pearson's Chi-squared test or Fisher's exact test as appropriate. All variables with *P* value < 0.2 in the univariate analysis were included for final multivariate analysis. Multiple logistic regression analysis was used to identify significant independent factors associated with surgery under 48 hours after adjusting for confounding factors such as age, gender, hypertension, diabetes, and dyslipidemia. The Wald test was computed on each predictor to determine which predictors were significant. Adjusted odds ratio and 95% confidence interval for the adjusted odds ratio were reported.

Receiver operative curve (ROC) analysis was used to identify “optimal” cutoff point for* IGR2* which best separates patients undergoing DHC surgery ≤ 48 versus > 48 hours with respect to outcome. ROC analysis indicated IGR2 of >7.5 ml/hr as an optimal cutoff point with sensitivity of 70% and specificity of 63% and area under the curve was 0.764. A comparison of demographics, risk factors, and clinical characteristics was made between favorable [mRS ≤ 4] and unfavorable [mRS > 4] outcomes at three months. Multiple logistic regression analysis was used to identify significant independent factors associated with favorable [mRS ≤ 4] and unfavorable [mRS > 4] outcomes after adjusting for confounding factors such as age, gender, hypertension, diabetes, and dyslipidemia. A *P* value < 0.05 (two-tailed) was considered statistically significant.

## 5. Results

Two hundred and thirteen patients were selected for DHC based on the above criteria. One hundred and forty-six patients underwent DHC and 67 patients initially selected for surgery were not operated on [*n* = 19 who refused surgery died and *n* = 48 stabilized without further deterioration and treating physician/surgeon decided not to operate on them]. Nine patients were excluded from the DHC analysis (incomplete data, *n* = 2; hemorrhage with ventricular extension, *n* = 4; hemorrhage deemed to have caused acute worsening (PH II), *n* = 3). The final analysis included 137 patients who underwent DHC (UAE, *n* = 59; Qatar, *n* = 45; Pakistan, *n* = 33).

### 5.1. Demographic, Clinical, and Imaging Variables and Outcomes (Table 1)

Fifty-four patients with a mean age of 47.8 ± 10.6 years underwent DHC at or less than 48 hours from the onset of symptoms, while 83 patients with a mean age of 47.9 ± 11.3 years were operated on more than 48 hours from symptom onset (Table 1). There was no difference in the prevalence of risk factors, GCS at herniation, use of antiedema medication, and admission NIHSS [*P* = 0.406] between the two groups. Although the mean final infarct volume [MIV], first infarct volume [IV1], and second infarct volume [IV2] were comparable between two groups, more patients who had MCA stroke with additional infarcts [MCA+] ACA/PCA had surgery at or less than 48 hours. Mean time to DHC was 51.33 hours (range: 12 to 312 hours: less than 24 hours, 15 (10.9%); 24–less than 48 hours, 39 (28.5%); 48–72 hours, 44 (32.1%); and more than 72 hours, 39 (28.5%)). IGR was calculated to determine a value of IGR that led to early versus late surgery. Both IGR1 and IGR2 were nearly double in patients with early surgery.

### 5.2. Outcome (Table 1 and Figures 1 and 2)

**Figure 1:**
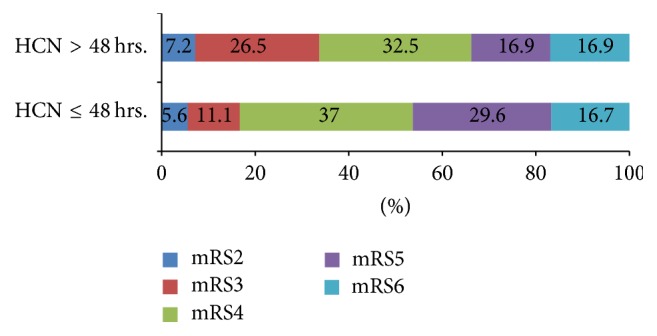
Outcome at three months with DHC more than 48 hours versus at or less than 48 hours [*P* = 0.140].

The functional outcome analyzed as mRS 0–4 versus mRS 5-6 did not show any significant difference in this split between early and late operated on patients [*P* = 0.140] and mortality [*P* = 0.975] at three months. More patients survived with mRS ≤ 3 with DHC more than 48 hours and severe disability [mRS 4-5] was more in patients operated on under 48 hours (*P* = 0.140; Figure 1) but the difference was not statistically significant.

A subgroup analysis according to time of DHC [less than 24, 24–48, 48–72, and more than 72 hours] and outcome at three months did not show any significant differences in outcome (*P* = 0.747).

Twenty-three patients died [16.8%]. There was no difference in mortality with early or late DHC [*P* = 0.975]. There was no statistical difference in subgroups by time and mortality (*P* = 0.973). Of the patients who died, 19/23 (82.60%) had MCA with additional infarcts (*P* < 0.001).

### 5.3. Multivariate Analysis

*Outcome (Table 2).* Multiple logistic regression analysis showed that age ≥ 55 [5.78: 2.14–15.64, *P* = 0.001], MCA with additional infarction [6.8: 2.88–16.48, *P* < 0.001], septum pellucidum deviation ≥1 cm [2.52: 1.02–6.20, *P* = 0.045], and uncal herniation (3.95: 1.48–10.49, *P* = 0.006) were independently associated with an unfavorable outcome at three months. Higher IGR2 was associated with poor outcome in univariate but not in multivariate analyses.

### 5.4. Time to Surgery (Table 3)

In the “best” multivariate model using IGR2 > 7.5 ml/hr [based on ROC analysis], patients with IGR2 of more than 7.5 ml/hr were 4 times more likely to have surgery at or less than 48 hours. Similarly, patients with MCA with additional infarction and patients with temporal lobe involvement were three times more likely to be operated on under 48 hours. IGR2 > 7.5 ml/hr, MCA with additional infarction, and temporal lobe involvement were independently associated with surgery at or less than 48 hours after adjusting for age, gender, hypertension, diabetes, and dyslipidemia (Table 3).

## 6. Discussion

**Figure 2:**
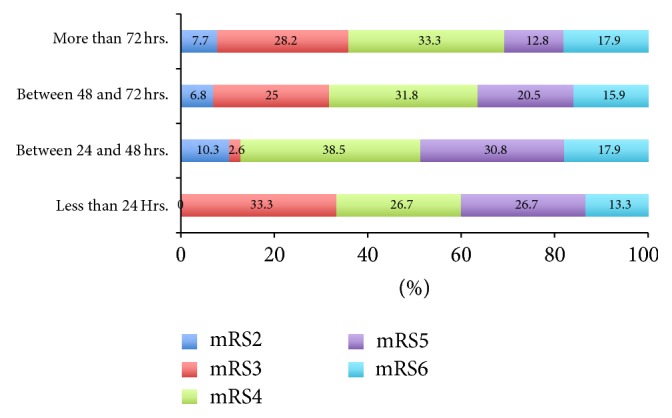
Outcome at three months with DHC stratified by time to surgery (*P* = 0.747).

We report on the largest pooled DHC dataset to date of DHC in MMCA stroke patients operated on after 48 hours of stroke onset from three different countries. Our data shows that patients operated on after 48 hours had similar outcomes compared to those operated ≤ 48 hours (Table 1 and Figure 1). Similar to the results of HeADDFIRST [12], requiring significant mass effect within 96 hours of stroke onset for randomization, we did not find an increase in disability or mortality with delayed surgery [after 48 hours]. No difference was noted in the demographics, risk factors, clinical severity of stroke, and infarct volume amongst the two groups (Tables 1 and 2). The subgroup analysis by time to surgery confirms our main results demonstrating that time to surgery was not a significant factor affecting the outcome (Figure 2).

The number of patients in the randomized trials treated within 48 hours is still small [*n* = 58], and a valid comparison with patients treated later [*n* = 11] is not possible. Only a systematic review by Gupta et al. included 51 patients operated on later than 48 hours [6]. The rate of severe disability in current study was higher, at 66.3%, when using mRS ≤ 3 compared with 55% in the systematic review. Most patients in our study survived with mRS of 4 [at or less than 48 hours, 37.0%, versus more than 48 hours, 32.5%] similar to the results of an updated meta-analysis [19]. This could be due to the inclusion of older patients [>55 years] with nearly six times the odds of poor outcome in the current study similar to the previous report [20]. We choose the 55 years of age cutoff based on the published data about stroke in patients from South Asia and the Gulf States occurring at a significantly younger age than in Caucasian patients [21, 22]. In our cohort, patients with uncal herniation were four times more likely to have poor outcome and poor outcome was 2.5 times more likely if there was subfalcine herniation [septum pellucidum deviation more than 1 cm] (Table 2). In the study by Mori et al., better outcome was observed if patients were operated on before signs of herniation appear, though the time to surgery was more than 48 hours [mean time to surgery: 2.78 days ± 0.21 versus 2.48 days ± 0.87] in both groups [23]. In the systematic review of uncontrolled studies, presence of signs of herniation before surgery did not affect the outcome; however, data on signs of herniation before surgery was available in only 59% of patients and missing data may have biased their results [6]. Our data shows that patients with MCA and additional infarction were seven times more likely to have a poor functional outcome [mRS 5-6]. Not only was the outcome worse, but also the chance of early DHC was 3.5 times more likely (Table 3). Moreover, DHC is contraindicated in patients with MCA with additional stroke in the Swiss recommendations for decompressive surgery for MMCA strokes [24].

The low mortality observed in the conservatively treated patients randomized after 48 hours (36% in HAMLET and 40% in HeADDFIRST) compared with patients randomized less than 48 hours (78% in HAMLET, 53% in DESTINY, and 78% in DECIMAL) points to a possibility of two different MMCA stroke groups, that is, the group of those that deteriorate more rapidly and hence were operated on early and the group of those that deteriorate slowly and hence were operated on late. Uhl et al. also noticed two groups in their cohort, early surgery and late surgery, based on more rapid decline of the GCS score (0.21 versus 0.08 GCS points/hour), though the median GCS score before surgery was not significantly different between the two groups [7].

In the absence of randomized trials, the published literature about the timing of surgery is conflicting. The published literature about delayed surgery has a number of shortcomings including small number of patients [ranging from 5 to 13], wide range of surgical timings [49 to 459 hours], and variable outcome. [4, 7–9, 11–16, 25–30]. Only HAMLET and HeADDFIRST trials allowed randomization up to 96 hours [4, 25]. The number of patients reported is too small to draw any conclusion about timing of surgery and its impact on outcome.

One of the goals of the present study was to identify factors contributing to early [less than 48 hours] surgery. In our analysis, the infarct volume of patients who underwent DHC less or more than 48 hours was not significantly different (Table 1 and Figure 1). Our data shows that the time difference to reach infarct volume on second CT [no statistical difference between infarct volumes] was 31.5 hours between early surgery and late surgery. Hence, the infarct growth rate was nearly double in patients who had DHC done under 48 hours (Table 1). Wheeler et al. in a substudy of DEFFUSE 2 have shown higher initial infarct growth rate in patients with malignant profile [31]. With an IGR cutoff of 7.5 ml/hr, our patients were four times more likely to have DHC at or less than 48 hours (Table 2). Our data is supported by previously reported penumbral loss rate of 8.9 ml/hr without collateral flow [32].

The variations observed in IGR suggest factors more important than time in determining the optimal time for DHC, such as collateral circulation failure and development of vasogenic edema. The collaterals have a major impact on penumbral evolution and support and failure is associated with infarct growth [33]. Presence of good collaterals can extend the time treatment window of acute stroke by slowing down the loss of penumbral tissue. A wide range of infarct growth rates also suggests a wide variation in collateral flow and penumbral loss as previously reported [32]. Therefore, the patients with slow penumbral loss will have a slower infarct growth rates and may benefit from treatment beyond the current less than 48 hours limit. Therefore, patients undergoing surgery after 48 hours from stroke onset should not be pooled with patients operated on before 48 hours. The convincing results of the European trials and their pooled analysis should be applied to the patients with rapid IGR [more than 7.5 ml/hr] operated on at or less than 48 hours.

Our study has several limitations including the retrospective nonrandomized nature. Another limitation relates to the assessment of the IGR on CT scans. We presumed that CT changes were present when the stroke symptoms began. It is possible that the hypodensity developed at a later time interval; hence, the first IGR may have a different value. However, the IGR on second CT did not show any significant difference presuming that infarct growth is linear in the acute stage [31, 34]. We observed relatively large variations in standard deviation of the mean IGR. The wide variation in IGR has been observed by others as well and likely reflects the genetic variation in collaterals and the rate of collateral failure [31, 32, 35]. The criteria for surgery may have differed between physicians/surgeons and centers. While most patients were operated on at the appearance of herniation signs, others were operated on at the deterioration of level of consciousness; thus a selection bias cannot be excluded. Finally, a major limitation is the short term (3-month) follow-up and type of rehabilitation received. The difference in rehabilitation facilities, more organized in Qatar compared to other centers, could be another factor affecting the outcome. Because of most significant functional recovery happening within the first six months after stroke, a minimal observation period of 6 months is recommended [36]. Since most expatriates leave the country [Qatar and UAE] after treatment, long-term follow-up was not available.

In conclusion, we present the outcomes in the largest pooled series of patients undergoing DHC after 48 hours and show that the functional outcome and mortality were comparable to patients operated on under 48 hours. Our data identifies IGR, temporal lobe involvement, and MCA with additional infarct as independent predictors of early surgery in MMCA patients. Our study has the potential to improve the early selection of patients and may guide clinical judgment in decision-making regarding time to DHC. DHC should not be time-barred and patients can still benefit beyond 48 hours.

## Figures and Tables

**Table 1:** Demographic, clinical, radiological imaging, and surgical data in relation to the time of surgery.

Factors	Total *n* = 137	HCN ≤ 48 (*n* = 54, 39.4%)	HCN > 48 (*n* = 83, 60.6%)	*P* value
Age in years	47.8 ± 11.0	47.8 ± 10.6	47.9 ± 11.3	0.966
<55 years	104 (75.9)	41 (75.9)	63 (75.9)	0.998
≥55 years	33 (24.1)	13 (24.1)	20 (24.1)	
Gender				
Male	111 (81.0)	41 (75.9)	70 (84.3)	0.220
Female	26 (19.0)	13 (24.1)	13 (15.7)	
Hypertension	63 (46.0)	24 (44.4)	39 (47.0)	0.770
Diabetes	47 (34.3)	22 (40.7)	25 (30.1)	0.201
Dyslipidemia	50 (36.5)	22 (40.7)	28 (33.7)	0.405
Coronary artery disease	30 (21.9)	10 (18.5)	20 (24.1)	0.440
Affected side				
Left	64 (46.7)	25 (46.3)	39 (47.0)	0.937
Right	73 (53.3)	29 (53.7)	44 (53.0)	
Herniation time (from onset)				
<24 hours	29 (21.2)	25 (46.3)	4 (4.8)	<0.001
24–<48 hours	51 (37.2)	29 (53.7)	22 (26.5)	
48–<72 hours	30 (21.9)	0	30 (36.1)	
≥72 hours	27 (19.7)	0	27 (32.5)	
GCS at herniation^(118)^	7.57 ± 2.36	7.6 ± 2.5	7.6 ± 2.3	0.957
Pupillary abnormality	27 (19.7)	10 (18.5)	17 (20.5)	0.778
Bilateral Babinski	83 (60.6)	34 (63.0)	49 (59.0)	0.646
Comatose	83 (60.6)	32 (59.3)	51 (61.4)	0.798
Uncal herniation	92 (67.2)	43 (79.6)	49 (59.0)	0.012
Mean time of first CT	7.71 ± 8.6	6.4 ± 7.5	8.5 ± 9.2	0.293
Mean time of second CT	45.15 ± 40.7	25.8 ± 10.1	57.3 ± 47.5	<0.001
Mean CT time for maximum infarct volume	58.1 ± 55.78	28.9 ± 13.4	76.9 ± 64.2	<0.001
Mean infarct volume on 1st CT	117.4 ± 108.5	123.1 ± 114.6	113.7 ± 104.9	0.624
Mean infarct volume on 2nd CT	325.1 ± 131.5	349.5 ± 130.1	309.8 ± 130.9	0.094
Maximum infarct volume (cm^3^)	367.7 ± 119.4	371.8 ± 125.3	365.0 ± 116.2	0.747
Infarct growth rate 1 (ml/hr)	10.3 ± 7.5	15.2 ± 8.1	7.1 ± 5.03	<0.001
Infarct growth rate 2 (ml/hr)	9.7 ± 7.9	13.6 ± 8.7	7.2 ± 6.2	<0.001
Type of MCA infarction				
Without additional infarct	80 (58.4)	24 (44.4)	56 (67.5)	0.008
With additional infarct	57 (41.6)	30 (55.6)	27 (32.5)	
Temporal lobe involved	86 (62.8)	41 (75.9)	45 (54.2)	0.010
SP displacement (last CT)	1.01 ± 0.37	0.89 ± 0.38	1.1 ± 0.35	0.003
>1 cm	81 (59.1)	26 (48.1)	55 (66.3)	0.035
≤1 cm	56 (40.9)	28 (51.9)	28 (33.7)	
Vascular occlusion present	114 (83.2)	46 (85.2)	68 (81.9)	0.618
Type of vessel occluded				
MCA	81 (71.1)	27 (58.7)	54 (79.4)	0.056
MCA/ACA	12 (10.5)	7 (15.2)	5 (7.4)	
ICA/MCA/ACA	21 (18.4)	12 (26.1)	9 (13.2)	
Prognosis at 90 days				
MRS 0–4	84 (61.3)	29 (53.7)	55 (66.3)	0.140
MRS 5-6	53 (38.7)	25 (46.3)	28 (33.7)	

*P* value has been measured using Mann–Whitney *U* test. Results are expressed as mean ± standard deviation, median (interquartile range), and number (percentage). *P* value has been calculated using binary logistic regression Wald test. CT, computer tomography; SP, septum pellucidum; MIV, maximum infarct volume; cm^3^, cubic centimeters.

**Table 2:** Multivariate analysis of factors impacting outcome [mRS ≤ 4 versus >4].

Factors	Adjusted odds ratio	95% CI for adjusted odds ratio	*P* value
Age ≥ 55	5.78	2.14–15.64	0.001
MCA with additional infarct	6.8	2.88–16.48	0.001
Uncal herniation	3.95	1.48–10.49	0.006
Septum pellucidum deviation ≥1 cm	2.52	1.02–6.20	0.045

CI: confidence interval. *P* value has been calculated using binary multiple logistic regression Wald test.

The dependent variable was the functional outcome mRS 0–3 versus 5-6.

**Table 3:** Multivariate analysis of factors impacting time to surgery, adjusted for Age, gender, HTN, DM, and dyslipidemia.

Factors	Adjusted odds ratio	95% CI for adjusted odds ratio	*P* value
Infarct growth rate 2 > 7.5 ml/hr	4.1	1.85–9.1	0.001
MCA with additional infarct	3.5	1.5–8.1	0.004
Temporal lobe involved	3.1	1.3–7.6	0.012

CI: confidence interval. *P* value has been calculated using binary multiple logistic regression Wald test. ml/hr, milliliter/hour. Dependent variable was time to surgery <48 or >48 hours.
